# Horny Growth from the Head in the Human Subject

**Published:** 1849-07

**Authors:** 


*Horny Growth from the Head in the Human Subject.*—The following curious example of the development of a horn from the head in the human subject, has recently fallen under the notice of Dr. Blasbury, and detailed in *Casper's Wocheus Chrift.* The individual in whom this occurred was an old man, eighty-four years of age. The horn sprang from the right temple close to the outer angle of the orbit; it was about three inches in length, stood straight out from the head, and gradually tapered to a point from the base to the extremity. The diameter at the base was an inch and a half; the point was slightly crooked. It was said to have originated in a kind of warty growth, and to have occupied about a year and a half in attaining its full size. It afforded him no pain or uneasiness. An ignorant quack having persuaded the man that he could remove it without pain, proceeded to apply some corrosive material to the base of the horn, by which its removal was indeed shortly effected, but in its place there was left a painful and extensive ulcerated surface, of a malignant aspect, which continued to spread, and in four months caused the old man's death.

